# Retained Intrauterine Device (IUD): Triple Case Report and Review of the Literature

**DOI:** 10.1155/2018/9362962

**Published:** 2018-12-05

**Authors:** Mon-Lai Cheung, Shadi Rezai, Janelle M. Jackman, Neil D. Patel, Basem Z. Bernaba, Omid Hakimian, Dilfuza Nuritdinova, Catherine L. Turley, Ray Mercado, Takeko Takeshige, Sudha M. Reddy, Paul N. Fuller, Cassandra E. Henderson

**Affiliations:** ^1^Department of Obstetrics and Gynecology, Southern California Kaiser Permanente, Kern County, 1200 Discovery Drive, Bakersfield, California 93309, USA; ^2^Department of Obstetrics and Gynecology, The Brooklyn Hospital Center, 121 DeKalb Avenue, Brooklyn 11201, USA; ^3^St. George's University, School of Medicine, St. George's, West Indies, Grenada; ^4^Department of Obstetrics and Gynecology, Lincoln Medical and Mental Health Center, 234 East 149th Street, Bronx, New York 10451, USA; ^5^Department of Obstetrics and Gynecology, Comprehensive Medical Care of the Bronx, 1931 Williamsbridge Road, Bronx, New York 10461, USA; ^6^Takeshige Medical, PO Box 121, Edgewater, New Jersey 07020, USA; ^7^Maternal and Fetal Medicine, Department of Obstetrics and Gynecology, Lincoln Medical and Mental Health Center, 234 East 149th Street, Bronx, New York 10451, USA

## Abstract

**Background:**

Throughout the world, intrauterine contraceptive devices (IUDs) are a frequently used, reversible, popular contraceptive method. They are usually placed without major complications. Uterine perforation is a rarely observed complication. Migration of the IUD to the pelvic/abdominal cavity or adjacent structures can occur after perforation. We present 3 cases of uterine perforation, possibly due to scarred myometrium associated with a cesarean delivery. We describe 3 perforations with IUDs lodged in the bladder serosa, the posterior cul-de-sac, and tissue adjacent to the cardinal ligament and external iliac artery.

**Cases:**

*Case  1*.  26-year-old, Gravid 4, Para 2113, nonpregnant female with a history of a cesarean delivery underwent placement of an IUD one year after an elective pregnancy termination, presenting with abdominal pain requesting removal of the IUD. On speculum, although the IUD strings were visualized, the IUD could not be removed. Sonogram imaging identified an empty endometrial cavity with the IUD in posterior cul-de-sac. The IUD was removed via laparoscopy.

**Case  2:**

34-year-old Gravida 5, Para 4004, at 27 weeks and 3 days gestation, female with history of two previous cesarean deliveries underwent a third cesarean after spontaneous rupture of membranes with comorbid chorioamnionitis. Reproductive history was significant for placement of an IUD that had not been removed or imaged during obstetrical sonograms. The clinical evaluation revealed that the IUD had been spontaneously expelled. On the fifth operative day, the patient is febrile with CT demonstrating the IUD penetrating the anterior surface of bladder. On cystoscopy the bladder mucosa was intact. The IUD was removed via laparotomy with repair of the bladder, serosa, and muscular layer.

**Case  3:**

26-year-old, Gravid 4, P3013, nonpregnant female with three previous Cesarean deliveries had an IUD in place. However, with the IUD in situ, the patient conceived and had a spontaneous abortion. After the spontaneous abortion, she presented to clinic to have the IUD removed due to pain that was present since placement. Although the IUD strings were visualized, attempts to remove it were unsuccessful. Imaging identified the IUD outside the uterine cavity. Palpation with a blunt probe laparoscopically revealed a hard object within the adhesion band, close to the cardinal ligament. As per radiology evaluation, IUD was embedded 1cm from the external iliac artery on the right side outside the uterus in the adnexal region. A multidisciplinary procedure with gynecologic-oncologist was scheduled for removal due to the high risk of perioperative bleeding.

**Conclusion:**

Patients in whom uterine perforation and IUD migration are suspected should have appropriate evaluation that includes transvaginal or transabdominal ultrasound or radiographs to confirm the position of the IUD, regardless of whether they are asymptomatic or present with symptoms. It is particularly important in the presence of a scarred uterus that imaging is used to identify the location of a missing IUD. The uterine scar of a cesarean may facilitate migration of the IUD. Cross sectional imaging, such as CT or MRI scan, may be needed to rule out adjacent organ involvement before surgical removal.

## 1. Background

Unintended pregnancy rates remain high at approximately 50% in the United States, particularly among adolescents, women who belong to racial/ethnic minority groups, and women in groups of lower socioeconomic status [1]. Thus, increasing the numbers of safe and effective choices for contraceptive methods, including long acting reversible contraceptive (LARC) such as intrauterine devices (IUD), to reduce the risk for unintended pregnancy is essential [1].

IUDs are a widely used method of contraception worldwide [2]. These devices are reliable, cost-effective, long acting, and reversible and can be used by a wide range of women [3, 4]. Their placement in the uterus is usually a simple and safe gynecological procedure [5]. The failure rate is approximately 0.2% for levonorgestrel releasing IUD and 0.8% for copper containing IUD, with typical use [1]. Complications seen with an intrauterine device are relatively uncommon but can be serious; examples include a lost IUD and uterine perforation [6]. The incidence of uterine perforation by an IUD is reported to be between 0.05 and 13 per 1000 insertions, with the results being potential serious complications [7]. After uterine perforation, the IUD can migrate into surrounding organs, particularly the bladder and sigmoid colon. Lost IUDs can be diagnosed and treated with minimally invasive procedures including hysteroscopy, endoscopy, and laparoscopy. If these are not able to retrieve the device, a more invasive procedure, such as exploratory laparotomy, should be considered.

During IUD-related abnormal uterine bleeding, no gross uterine or tissue pathology has been described other than for chronic inflammatory reaction of the endometrium. In half of the cases, the IUD was embedded, displaced within the uterine cavity, or migrated into the uterine wall, all of which may be a possible cause of the IUD-related abnormal uterine bleeding. For the detection and removal of the IUD with missing strings, partially embedded and perforated IUD, or retained broken parts, the hysteroscopic procedure is an invaluable method of choice [6, 8]. However, in a few cases, such as our cases, a more invasive procedure like laparoscopy is needed to remove the IUD. In this report, we present 3 cases of women, all with previous cesareans, who had ectopic IUDs associated with uterine perforation and IUD migration. In one case, the IUD was found in the serosa of the bladder wall; in the second case, it was found in the posterior cul-de-sac adjacent to the sigmoid colon; and in the third case, the IUD was embedded in tissue next to cardinal ligament 1cm from external iliac artery.

## 2. Presentation of Case 1

**Figure 1:**
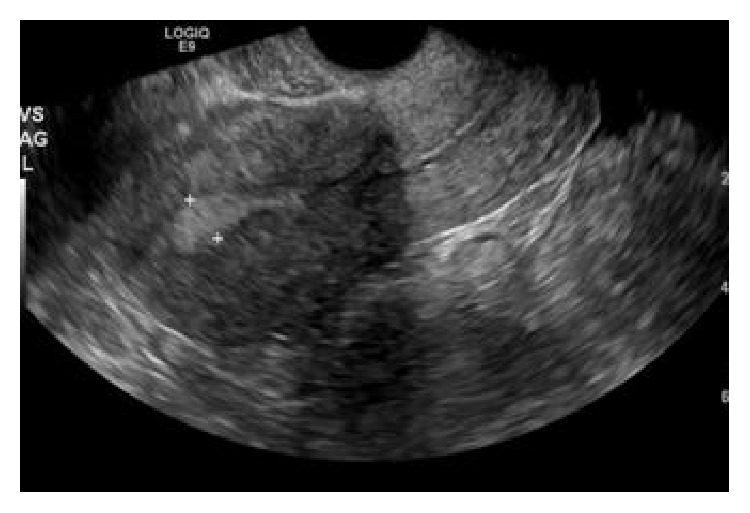
**CASE** **1**, pelvic ultrasound: empty uterine cavity with no evidence of intrauterine device (IUD).

**Figure 2:**
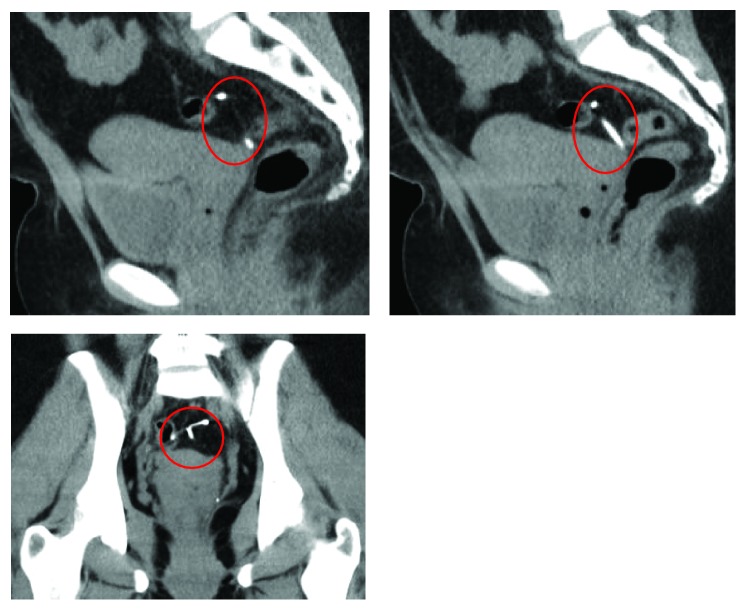
**CASE** **1**, pelvic CT: showing anteflexed anteverted uterus with IUD outside the uterus in the cul-de-sac.

**Figure 3:**
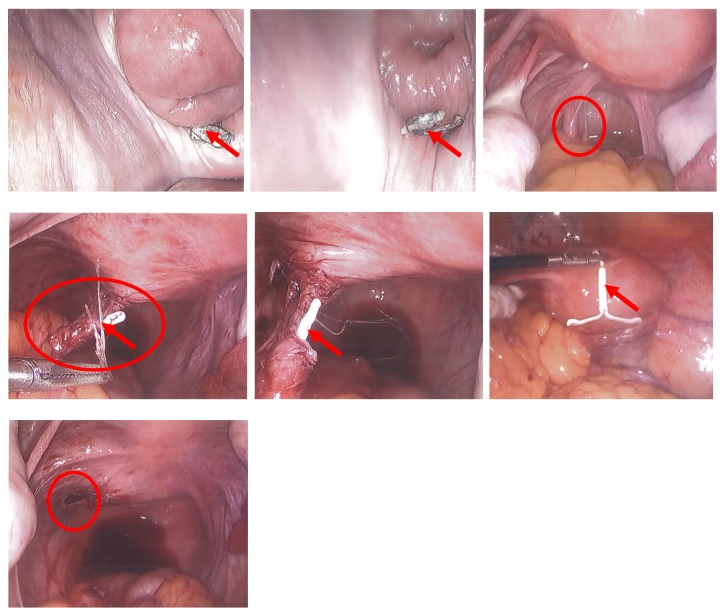
**CASE** **1**, laparoscopic removal of Mirena IUD from posterior cul-de-sac.

The patient is a 26-year-old, nonpregnant female, Gravid 4, Para 2113, with history of one previous cesarean (BMI 35.24 kg/m^2^), who presented with severe dysmenorrhea monthly since IUD placement and desires to switch to NuvaRing. The patient had Mirena IUD placed one year prior at outpatient clinic immediately after an elective termination of pregnancy. She presented with complaints of bleeding and pain. On initial speculum exam, the IUD strings were visualized, but attempts to remove it were unsuccessful. Additional speculum exam revealed strings through the vagina mucosa coming through the posterior fornix and not the cervix. Pelvic ultrasound (Figure 1) showed an empty uterine cavity with no evidence of IUD. Subsequent CT of the abdomen and pelvis (Figure 2) revealed T-shaped IUD, located midline within the posterior pelvic mesentery/cul-del-sac, superior to the posterior aspect of the anteverted uterus, and adjacent to the distal rectosigmoid colon. A diagnostic laparoscopy was performed (Figure 3) for retrieval and removal of malpositioned IUD and lysis of associated adhesions.

## 3. Presentation of Case 2

**Figure 4:**
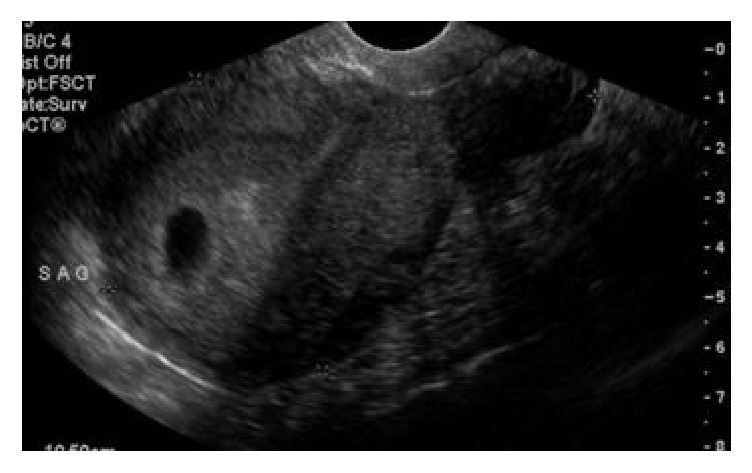
**CASE** **2**, pelvic ultrasound (5/7/15): there is no sonographic evidence of IUD (intrauterine contraceptive device) identified within the uterus. The uterus measures about 11.3 x 6.4 x 7.3-cm in size, with its cervical length measuring about 4.40-cm. A single live intrauterine gestation of 6 weeks of menstrual maturity with EDD of 12/31/2015.

**Figure 5:**
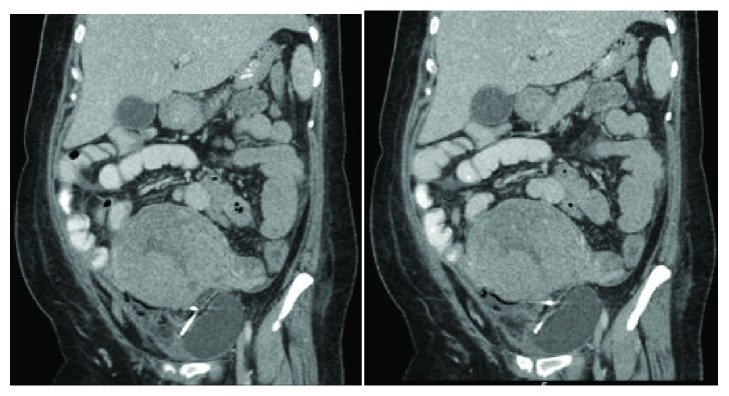
**CASE** **2** **: pelvic CT (10/15/2015) **that was done postpartum day 5: a CT scan revealed a displaced IUD with its tip appearing to have penetrated the lumen of the bladder. It was located in the anterior portion of the bladder near the space of Retzius.

**Figure 6:**
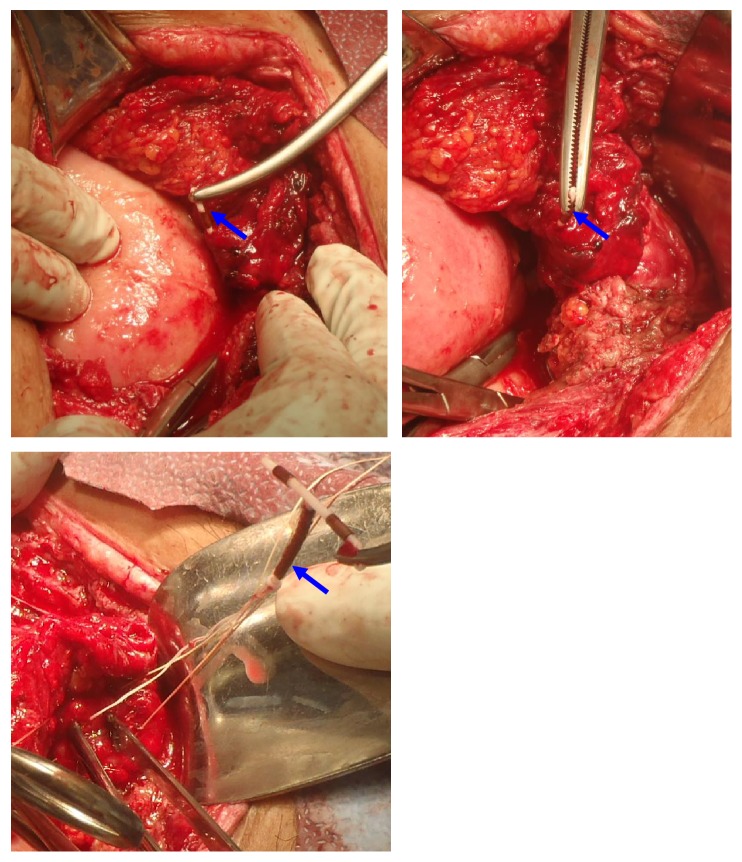
**CASE** **2**, exploratory laparotomy and removal of ParaGard IUD from the seromuscular layer of the bladder.

A 34-year-old female, Gravid 5, Para 4004, at 27 weeks and 3 days gestation, with two previous cesarean sections, presented with leakage of fluid per vagina. The patient has a past history of ParaGard IUD placement, which was not seen in initial obstetrics sonogram at 6 weeks of gestation (Figure 4). The clinical diagnosis was that the IUD had been expelled. Other obstetrics ultrasound during the pregnancy showed an intrauterine device. The patient was admitted with the findings of premature preterm rupture of membranes and suspected chorioamnionitis. A low transverse cesarean delivery section and bilateral tubal ligation were performed. On postoperative day 5, the patient experienced fevers with suspected intra-abdominal processes. A CT scan revealed a displaced IUD, with the tip penetrating the lumen of the bladder, located in the anterior portion of the bladder, near the space of Retzius (Figure 5). Cystoscopy confirmed that the IUD did not penetrate mucosa. Patient underwent laparotomy for removal of ParaGard IUD from the serosa and muscularis layer of the bladder (Figure 6) and the layers were then repaired.

## 4. Presentation of Case 3

**Figure 7:**
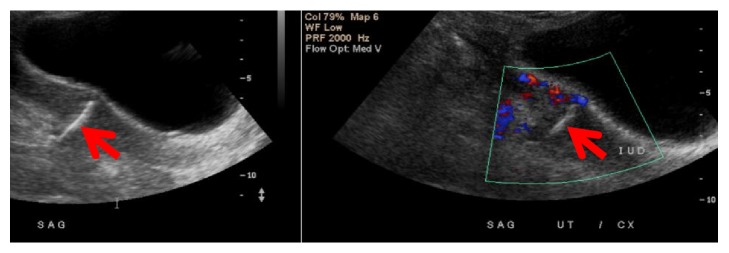
**CASE** **3**, ultrasound imaging showing the embedded IUD.

A 26-year-old nonpregnant, Gravid 4, Para 3013, with history of three previous cesarean deliveries, initially presented to women's clinic for IUD removal. She had an IUD placed 2 years ago, became pregnant 6 month after IUD placement, and had a first trimester spontaneous abortion at 8 weeks gestation. On initial presentation, the patient reported chronic right lower quadrant pelvic pain after IUD placement and desired to switch to oral contraceptive pills. An attempt was made to remove IUD in the women's clinic; IUD strings were visualized and grasped, but the device could not be removed. Initial sonogram (Figure 7) imaging showed the IUD running across the length of the cervix. Patient was then scheduled for diagnostic hysteroscopy and IUD removal in the operating room. Using the hysteroscope, IUD was visualized deep in the posterior uterine wall. Multiple attempts to remove the IUD with the use of hysteroscopic grasper were unsuccessful. In addition, with use of forceps under ultrasound guidance, the IUD could not be removed. Hysteroscopy was repeated after failed attempts of removal; however, the IUD strings were no longer seen. Intraoperative transvaginal ultrasound (TVS) showed the IUD in the posterior uterine wall in the lower segment with no free fluid in the cul-de-sac. The surgical procedure was aborted.

**Figure 8:**
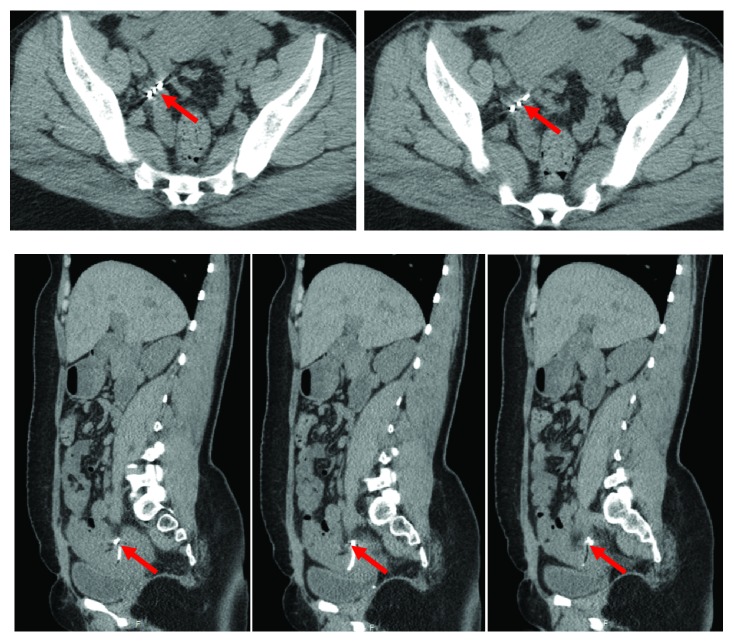
**CASE** **3**, CT imaging showing the embedded IUD.

Repeat ultrasound showed bilaminar, echogenic structure, consistent with an IUD, protruding through the posterior wall of the uterus, above the level of the cervix. CT of abdomen and pelvis (Figure 8) showed an anteverted uterus. The IUD appeared to extend vertically up towards the right from the level of the cervix, which is outside the uterine body. The patient was scheduled for diagnostic laparoscopy for IUD removal.

During the laparoscopy, examination showed a small cervix displaced anteriorly. No IUD was visualized intraperitoneally. Thick adhesion band was seen connecting the right posterior surface of the uterus to the right cardinal ligament in close proximity to uterus. Palpation with blunt probe laparoscopically revealed a hard object within the adhesion band close to cardinal ligament. Per radiology imaging, the IUD was embedded 1cm from the external iliac artery on the right side outside the uterus in adnexal region. The decision was made to stop procedure of the removal of IUD and refer the patient to a gynecologist-oncologist for further management. The patient received referral for schedule exploratory laparotomy in a secondary hospital with higher level of care.

## 5. Discussions

IUD is an accepted contraceptive method worldwide [9, 10]. IUDs are a popular method of reversible contraception as they have a high efficacy for fertility regulation, low risk, and low cost. [11–13]. Postinsertion follow-up and awareness of complications to assess for when the patient returns are important [12]. Common complications of IUD placement include, but are not limited to, abdominal or pelvic pain and abnormal bleeding, especially during the first few months after its insertion [13, 14]. Other adverse effects are expulsion, heavy bleeding, dysmenorrhea, unplanned pregnancy, and spontaneous abortion. In patients with a short-term and long-term history of IUD use, who present with pain, evaluation is essential to rule out IUD displacement, pelvic inflammatory disease, UTI, ectopic pregnancy, and uterine perforation. Expulsion, migration, or intrauterine displacement of the IUD leads to decreased contraceptive efficacy and necessitates removal of the defective IUD, with possible replacement to prevent contraception [14].

Two types of uterine perforation exist, and both are prone to serious device associated complications. Primary perforation may occur during insertion, which is typically associated with severe abdominal pain [15]. Secondary perforation is a delayed event, proposed to be due to gradual pressure necrosis of the uterine wall [11]. Once uterine perforation occurs, migration of the IUD outside the uterine cavity is a possible but also rare complication. Approximately 80% of IUDs are found in the peritoneal cavity after perforation. Migration into surrounding organs is a rare but serious complication after perforation [9, 10, 15]. Possible sites of migration include the omentum, rectosigmoid colon, peritoneum, bladder, appendix, small bowel, adnexa, and iliac vein [9, 10].

One should particularly be concerned for perforation if the IUD was inserted by an inexperienced user or is placed in an inappropriate position or the patient has a weakened uterine wall in proximity to insertion site, commonly occurring secondary to multiparity, cesarean section, or abortion [9, 10, 13, 16, 17]. In each of our cases, we have the factor of uterine wall susceptibility due to the history of previous cesarean sections. The device insertion may have affected the myometrium leading to uterine perforation and subsequent migration of the IUD. We hypothesize that in Cases 2 and 3, the fact that the patients conceived with IUD placement may have led to the secondary perforation, from increased uterine force exerted by the growing fetus.

In the case in which the IUD cannot be found, different methods can be utilized for finding and retrieving the device. In a case study by Cetinakaya et al., they found that 29 (52.7%) lost IUDs were located inside the uterine cavity, 23 (41.8%) were located outside the uterine cavity, and 3 (5.5%) were embedded in the myometrium. The most common extra-uterine location of lost IUDs was around the uterosacral ligaments [18]. IUD migration into adjacent organs leads to bowel obstruction, peritoneal perforation, appendicitis, vesical calculus formation, obstructive nephropathy, fistula formation, menouria, and intraperitoneal adhesions that can lead to infertility [11, 19]. Ultrasonography, x-ray, or CT is diagnostic to locate an IUD that has migrated [20, 21].

The World Health Organization recommends removing the migrated device as soon as possible [17, 22]. It is suggested that surgical removal should be considered even in asymptomatic patients once it has migrated out of the uterus [10, 23]. The recommendation is to use minimally invasive methods if possible, including hysteroscopy, cystoscopy, colonoscopy, or laparoscopy, depending on where the IUD is located. If the device is embedded in an organ such as the bladder or bowel, it is not recommended to remove it using minimally invasive methods; rather exploratory laparotomy should be performed. In a similar manner, like in the case discussed, if the device is embedded near a blood vessel or it is not completely visualized, more invasive methods are recommended by an experienced surgeon [15].

In one of the cases we presented, the IUD strings were visualized, but it could not be removed. Suspicion of an embedded device should be considered at this time. Minimally invasive procedures including the hysteroscopy and laparoscopy should be attempted first. Since the affected patient has a history of multiple cesarean deliveries, with presence of numerous adhesions, extra caution should be taken when exploring the abdomen for the device. As the IUD was not located, other approaches should be used to avoid further complications.

## 6. Conclusion

Many patients with uterine perforation and IUD migration may present with symptoms, but as many as 30% are asymptomatic. There is a need for prospective investigation on displaced/migrated IUDs, especially for women with a scarred uterus due to cesarean section or myomectomy; the weakened scar may lead to migration of the IUD. If a patient has a lost IUD and the threads are not visible during pelvic exam, preoperative vigilance, including transvaginal or transabdominal ultrasound or radiographs, should be obtained to confirm the position of the IUD. If IUD migration is suspected, cross sectional imaging, such as CT or MRI are recommended to rule out adjacent organ involvement before surgical removal. Hysteroscopy or laparoscopy should be considered prior to removing to avoid patient revisits to the operating room.
